# The significance of machine learning in neonatal screening for inherited metabolic diseases

**DOI:** 10.3389/fped.2024.1366891

**Published:** 2024-03-20

**Authors:** Xiangchun Yang, Shuxia Ding, Jianping Zhang, Zhuojie Hu, Danyan Zhuang, Fei Wang, Shanshan Wu, Changshui Chen, Haibo Li

**Affiliations:** ^1^ The Central Laboratory of Birth Defects Prevention and Control, Ningbo Women and Children's Hospital, Ningbo City, Zhejiang, China; ^2^ Ningbo Women and Children's Hospital, Ningbo, Zhejiang, China; ^3^ Department of Endocrinology, Ningbo Women and Children's Hospital, Ningbo, Zhejiang, China; ^4^ Department of Children’s Healthcare Clinic, Women and Children’s Hospital of Ningbo University, Ningbo, Zhejiang, China; ^5^ Paediatric Surgery Centre, Women and Children’s Hospital of Ningbo University, Ningbo, Zhejiang, China

**Keywords:** machine learning, neonatal screening, inherited metabolic diseases, diagnostic efficiency, carrier

## Abstract

**Background:**

Neonatal screening for inherited metabolic diseases (IMDs) has been revolutionized by tandem mass spectrometry (MS/MS). This study aimed to enhance neonatal screening for IMDs using machine learning (ML) techniques.

**Methods:**

The study involved the analysis of a comprehensive dataset comprising 309,102 neonatal screening records collected in the Ningbo region, China. An advanced ML system model, encompassing nine distinct algorithms, was employed for the purpose of predicting the presence of 31 different IMDs. The model was compared with traditional cutoff schemes to assess its diagnostic efficacy. Additionally, 180 suspected positive cases underwent further evaluation.

**Results:**

The ML system exhibited a significantly reduced positive rate, from 1.17% to 0.33%, compared to cutoff schemes in the initial screening, minimizing unnecessary recalls and associated stress. In suspected positive cases, the ML system identified 142 true positives with high sensitivity (93.42%) and improved specificity (78.57%) compared to the cutoff scheme. While false negatives emerged, particularly in heterozygous carriers, our study revealed the potential of the ML system to detect asymptomatic cases.

**Conclusion:**

This research provides valuable insights into the potential of ML in pediatric medicine for IMD diagnosis through neonatal screening, emphasizing the need for accurate carrier detection and further research in this domain.

## 1 Introduction

Inherited metabolic diseases (IMDs) comprise a group of genetic disorders, including amino acid, organic acid, and fatty acid disorders (1). The application of tandem mass spectrometry (MS/MS) in neonatal screening has revolutionized the early identification of IMDs by analyzing and interpreting amino acids and acylcarnitines (2). At present, MS/MS technology enables the screening of approximately 50 metabolites, facilitating the detection of over 20 IMDs (3). However, the comprehensive metabolite measurement involved in MS/MS screening comes with limitations, particularly in terms of false-positive (FPs) and false-negative results (FNs) (4–7). The consequences of such inaccuracies were significant, often subjecting families to unnecessary stress and healthcare costs or, conversely, delaying vital treatment.

With the advancement of science and technology, computational and machine learning (ML) methodologies provide a promising approach for analyzing high-dimensional data (8, 9). Recent applications of ML techniques have extended to neonatal screening for the diagnosis of IMDs, improving screening sensitivity and specificity (10, 11). ML techniques have the potential to expedite the diagnosis of IMDs. In a previous study conducted by our collaborative partners, 9 ML algorithms were employed to predict 16 IMDs (12). As the repository of screening and diagnostic data steadily accumulates, the ML system model has now acquired the capacity to prognosticate the presence of 31 IMDs (13). The present study aimed to conduct a comprehensive evaluation of the diagnostic efficacy of the ML system model using neonatal screening data from the Ningbo area (China) in clinical practice.

## 2 Materials and methods

### 2.1 Patients’ data

**Figure 1:**
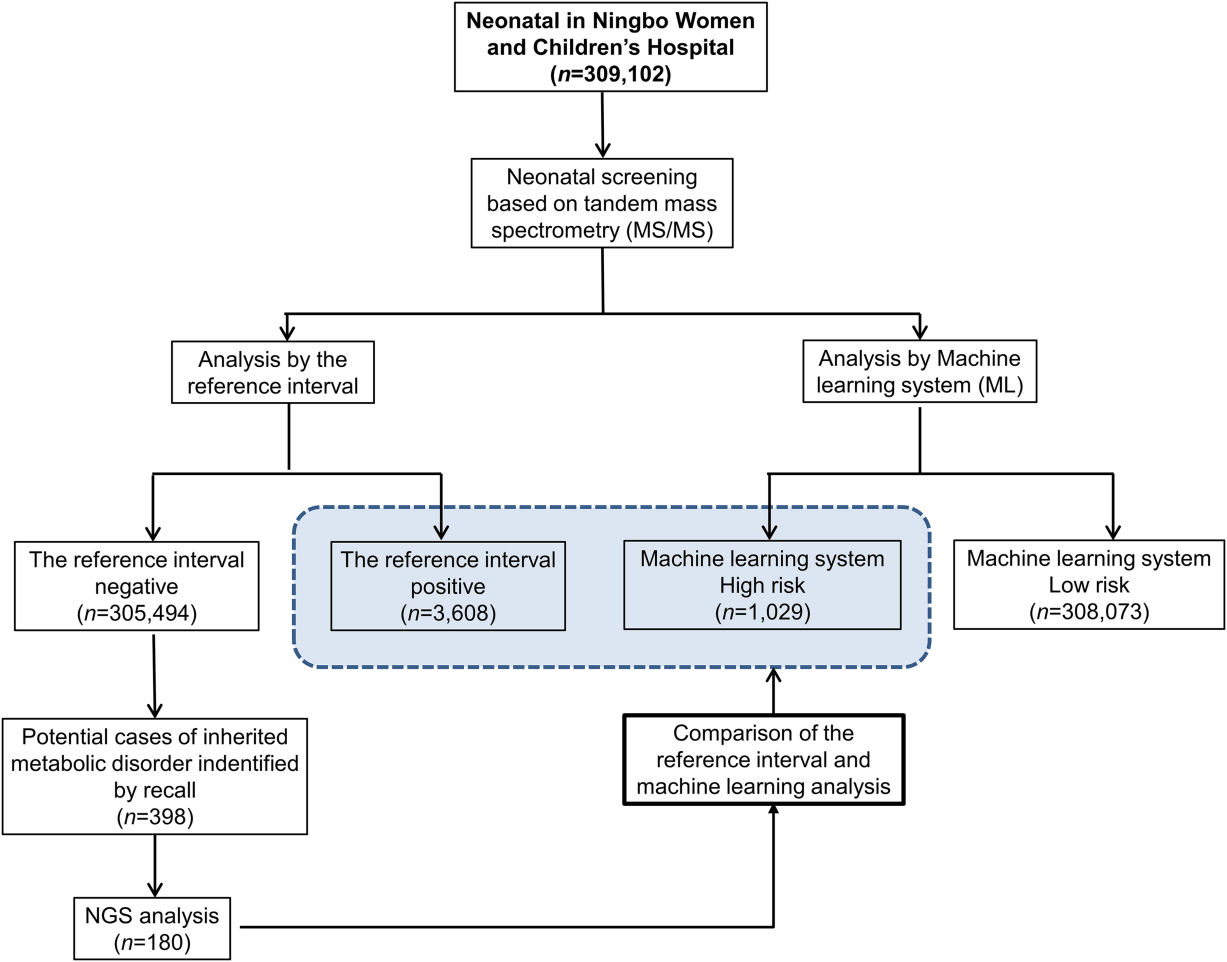
Workflow of the reference interval and machine learning for analyzing neonatal screening data of inherited metabolic diseases.

The study population consisted of 309,102 neonatal screening data collected from the Central Laboratory of Birth Defects Prevention and Control Affiliated with the Ningbo Women and Children's Hospital (Ningbo, China) between July 2014 and March 2020. In the large dataset of screening results, a total of 3,608 cases commenced recall procedures due to their initial positive screening results. Subsequently, 398 cases exhibited abnormal metabolic concentration or metabolite concentration ratios during secondary screening, indicating their potential as cases of IMDs. Among these, 180 cases underwent next-generation sequencing (NGS) to confirm IMDs, while the remaining 218 suspected cases did not proceed with NGS due to various reasons. These reasons include normal results from subsequent tandem mass spectrometry tests and urine organic acid tests, or parental refusal of NGS testing for their children. While these patients were included in the machine learning (ML) analysis, the lack of genetic testing reports for them precludes a definitive exclusion of disease presence. Therefore, our analysis focused solely on the diagnostic efficiency of cases with a clear genetic diagnosis, to ensure the integrity and reliability of our findings.The study flowchart is depicted in Figure 1.

### 2.2 MS/MS analysis

Quantification of amino acids and acylcarnitines in dried blood spots (DBS) was performed using the Xevo TQD tandem mass spectrometers (Waters Corp., Milford, MA, USA) in conjunction with the NeoBase Non-derivatized MSMS kit (PerkinElmer, Helsinki, Finland). The analysis comprehensively included 11 amino acids, encompassing a wide range of metabolites associated with the investigated amino acid disorders. In addition, 31 fatty acids (acylcarnitines) and succinylacetone were incorporated into the analysis, as they hold relevance to organic acidemias and fatty acid oxidation defects. Integral to our analysis, reference intervals for each metabolite were defined within the 0.5th to 99.5th percentile range, using a nonparametric ranking to address the non-normal distribution of our dataset. This careful selection ensures our screening's precision, with intervals refined as our sample pool expands, enhancing diagnosis accuracy by reducing false positives and negatives. The detailed information regarding the analyzed metabolites and their corresponding reference intervals can be found in Table S1.

### 2.3 Genetic testing and bioinformatics analysis

Genomic DNA was extracted from DBS or peripheral blood obtained from patients using the OMEGA Genomic DNA Extraction Kit (OMEGA Biotech, United States). Subsequently, targeted sequencing was conducted using the basic edition panel of IMDs (Genuine Diagnostic Laboratory, Hangzhou, China) to detect 94 genes, including *SLC22A5*, *PAH*, *PTS*, *MUT*, and other relevant genes. Target regions’ sequences were enriched through multiple probe hybridizations using the Agilent SureSelect Human Exon Sequence Capture Kit. Following enrichment, capture products were purified using Agencourt AMPure XP beads (Beckman Coulter). After purification and quality testing, the sequencing libraries were quantified using the Illumina DNA standard and Primer Premix Kit (Kapa), and subsequently subjected to massively parallel sequencing using the Illumina MiSeq platform. All potentially pathogenic variants were validated through Sanger sequencing utilizing specific primers. Polymerase chain reaction (PCR) conditions followed TaKaRa LA PCRTM Kit Ver.2.1 (TaKaRa). The trans status of all compound heterozygous variants was determined. Identified variants underwent scrutiny against databases such as the Human Gene Mutation (HGMD) Database, ClinVar, ExAC consortium, gnomAD, 1,000 Genome Project database, the laboratory's internal database (∼20,000 mutations), and relevant literature. Novel missense variants were further assessed for potential pathogenicity using tools integrated into VarSome, including SIFT, PolyPhen-2, and MutationTaster. Variant classification followed the standards and guidelines set forth by the American College of Medical Genetics and Genomics (ACMG).

### 2.4 Data collection and standardization for ML system model

a. *Laboratory Background Information:* This category covered various elements, including instrument models, types of reagents (commercial or self-made), types of quality control products, laboratory quality control rules, sizes of perforated blood spots, types of filter paper, cold chain transportation of blood samples, types of blood collection needles, succinylacetone treatment methods, normal population ranges, and interpretation rules.
b. *Quality Control Data:* This section comprised information such as quality control numbers, types of quality control, quality control batch numbers, amino acid internal standard batch numbers, acylcarnitine internal standard batch numbers, factory times, experimental times, and test values for each quality control analyte.
c. *Screening Test Data:* Included data on screening numbers, mother's ages, gestational ages, gravidities, parities, hyperthyroidism presence, anti-thyroid drug use, breastfeeding methods, milk consumption, native places (provinces and cities), places of residence (provinces and cities), baby genders, birth dates, birth weights, initial screening conclusions, recall review conclusions, sample numbers, screening times, blood collection dates, delivery dates, experimental dates, experimental methods (derivative or non-derivative), quality control numbers, and detection concentrations for each analyte.
d. *Positive Case Data:* This category covered screening numbers, confirmed diseases, urine organic acid tests, blood ammonia tests, blood gas analyses, blood routines, liver function tests, vitamin B12 tests, imaging examinations, genetic test information.
e. Standardized median multiple (multiple of the medium, MoM) methods were applied in which the median of the original concentration divided by the biochemical indicators was applied to the detection indicators to eliminate the influence of regional and laboratory differences. We then trained the disease model by combining the MoM, gestational week, neonatal blood collection interval, neonatal weight, and corresponding IMDs.

### 2.5 ML system model

The ML framework utilized in this study has been previously established and published by our collaborative partners, as detailed in the prior research (12, 13). This ML system model for IMDs was crafted by Zhejiang Biosan Biochemical Technologies Co. Ltd. To facilitate the training and testing of this model, an extensive dataset encompassing over 3.67 million neonatal screening data and 3,000 confirmed cases were collected from 31 hospitals between February 2010 and September 2019. Nine pertinent classification algorithms, comprising logistic regression, linear discriminant analysis, decision trees, random forest, extremely randomized trees, gradient boosting, adaptive boosting, support vector machine, and k-nearest neighbors, were systematically applied to analyze 31 IMDs. The detailed list of IMDs was presented in Table 1.

**Table 1:** The list of inherited metabolic disorders in machine learning system model.

Abbreviations	IMDs (OMIM code)	Gene
PAHD	Phenylalanine hydroxylase deficiency (#261,600)	*PAH*
B4HD	Tetrahydrobiopterin deficiency (#261,600)	*PTS,GCH1,QDPR, PCBD1*
PCD	Primary carnitine deficiency (#212,140)	*SLC22A5*
MMA	Methylmalonic acidemia (#251,000, #251,100,#251,110,#613, 646, and #614, 265)	*MUT,MMAA,MMAB,*
MMA-HCY	Methylmalonic aciduria combined with homocystinuria (#277,400, #277,410, #277,380, #309,541 and #614, 857)	*MMACHC,MMADHC, LMBRD1, ABCD4,*
PA	Propionic acidemia (#606,054)	*PCCA, PCCB*
SCADD	Short-chain acyl-CoA dehydrogenase deficiency (#201,470)	*ACADS*
IBDD	Isobutyryl-CoA dehydrogenase deficiency (#611,283)	*ACAD8*
3-MCCD	3-methylcrotonyl-CoA carboxylase deficiency (#210,200 and #210,210)	*MCCC1, MCCC2*
3-HMGD	3-hydroxy-3-methylglutaryl CoA lyase deficiency (#246,450)	*HMGCL*
BKD	β-ketothiolase deficiency (#203,750)	*ACAT1*
HCSD	Holocarboxylase synthetase deficiency (#253, 270)	*HLCS*
NICCD	Neonatal intrahepatic cholestasis citrin deficiency (#605,814)	*SLC25A13*
CIT-I	Citrullinemia type I (#215,700)	*ASS1*
ASA	Argininosuccinic aciduria (#207,900)	*ASL*
HMET	Hypermethioninemia (#250,850)	*MAT1A*
HCY	Homocystinuria (#236,200)	*CBS, CTH, MTHFR*
GA-I	Glutaric acidemia I (#231,670)	*GCDH*
IVA	Isovaleric acidemia (#243,500)	*IVD*
2-MBDD	2-methylbutyryl-CoA dehydrogenase deficiency (#610,006)	*ACADSB*
MCADD	Medium-chain acyl-CoA dehydrogenase deficiency (#201,450)	*ACADM*
VLCADD	Very-long-chain acyl-CoA dehydrogenase deficiency (#201,475)	*ACADVL*
GA-II	Glutaric acidemia II (# 231,680)	*ETFA, ETFB, ETFDH*
MSUD	Maple syrup urine disease (#248,600)	*BCKDHA, BCKDHB, DBT*
CPT-I	Carnitine palmitoyltransferase I deficiency (#255,120)	*CPT1A*
HPRO	Hyperprolinuria (#239,500)	*PRODH*
HTYR	Tyrosinemia type I (# 276,700)	*FAH*
OTCD	Ornithine transcarbamylase deficiency (#311,250)	*OTC*
CPSD	Carbamoyl phosphate synthetase deficiency (# 237,300)	*CPS1*
GCE	Glycine Encephalopathy (# 605,899)	*AMT, GCSH, GLDC*
HARG	Argininemia (# 207,800)	*ARG1*

IMDs, inherited metabolic diseases; OMIM, online Mendelian inheritance in man.

The construction of the ML system model followed a structured progression:

a. *Indicator Construction and Selection*: Core indicators were selected using information gain and correlation coefficients, culminating in the creation of feature combinations.
b. *Model Selection and Training*: During the model training phase, the screening data was partitioned into training and test sets, adhering to an 8:2 ratio. Subsequently, the most proficient model was identified through the integration of various learning models, including random forest, gradient boosting, and artificial neural network algorithm.
c. *Model Evaluation*: Initially, a 100% identification rate for positive cases was successfully achieved by the model. Subsequently, the FP rates were compared to discern and select the optimal training model. Additionally, within the risk assessment process, risk predictions for test samples were conducted by the ML model, estimating the risk associated with samples affected by various inherited metabolic diseases. These predictions were systematically converted into scores ranging from 0 to 100 via a dedicated risk value mapping algorithm. Distinct risk assessment reference interval was established, guided by the risk score and the disease prevalence specific to each location.

Finally, we used our ML technique to design an easy-to-operate Web-based screening system for neonatal metabolic diseases. This system was specifically designed to assess the risk of specific IMDs in each screening dataset, where high- and low-risk cases were classified as positive and negative, respectively.

## 3 Results

### 3.1 Neonatal screening and children with positive diagnosis

In our research, a primary screening of 309,102 neonates led to the identification of 3,608 infants with positive screening results, constituting a positive rate of approximately 1.17%. Further investigation of this group uncovered 398 infants who presented abnormal results during the secondary screening phase, prompting considerations of IMDs. Consequently, NGS was employed in 180 of these cases, to confirm the presence of IMDs.

**Figure 2:**
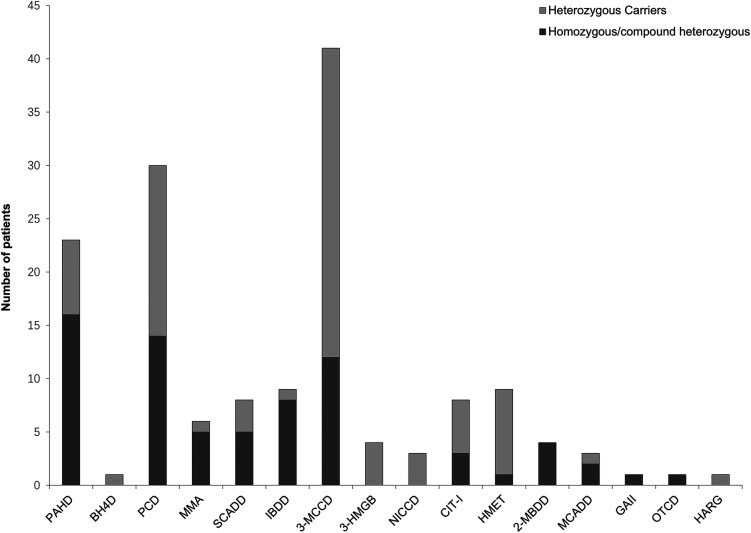
Number of patients with inherited metabolic diseases identified by next-generation sequencing. A total of 152 patients were confirmed to be positive for inherited metabolic diseases, covering a diverse spectrum of 16 types of inherited metabolic diseases. ML, machine Learning; TP, true-positive; PAHD, phenylalanine hydroxylase deficiency; BH4D, tetrahydrobiopterin deficiency; PCD, primary carnitine deficiency; MMA, methylmalonic acidemia; SCADD, short-chain acyl-CoA dehydrogenase deficiency; IBDD, Isobutyryl-CoA dehydrogenase deficiency; 3-MCCD, 3-methylcrotonyl-CoA carboxylase deficiency; 3-HMGD, 3-hydroxy-3-methylglutaryl CoA lyase deficiency; NICCD, neonatal intrahepatic cholestasis caused by citrin deficiency; CIT-I, citrullinemia type I; HMET, hypermethioninemia; 2-MBDD, 2-methylbutyryl-CoA dehydrogenase deficiency; MCADD, medium-chain acyl-CoA dehydrogenase deficiency; GA-II, glutaric acidemia type II; OTCD, ornithine transcarbamoylase deficiency; HARG, argininemia; PHE, phenylalanine; CIT, citrulline; MET, methionine; ARG, arginine; C0, free carnitine; C3, propionylcarnitine; C4, butyrylcarnitine; C5OH, 3-hydroxy (OH) isovalerylcarnitine; C6, hexanoylcarnitine; C8, octanoylcarnitine; C10, decanoylcarnitine; C12, dodecanoylcarnitine; C14, myristoylcarnitine; C14:1, myristoleylcarnitine; C14:2, tetradecadienoylcarnitine.

The results showed that 152 out of 180 patients who underwent NGS testing were positive for IMDs, of which 72 were homozygous or compound heterozygous mutations and the other 80 were heterozygous carriers. The results of our neonatal screening displayed a broad spectrum of metabolic disorders, encompassing 16 types of IMDs. These included phenylalanine hydroxylase deficiency (PAHD, 23 cases), tetrahydrobiopterin deficiency (BH4D, 1 case), primary carnitine deficiency (PCD, 30 cases), methylmalonic acidemia (MMA, 6 cases), short-chain acyl-CoA dehydrogenase deficiency (SCADD, 8 cases), Isobutyryl-CoA dehydrogenase deficiency (IBDD, 9 cases), 3-methylcrotonyl-CoA carboxylase deficiency (3-MCCD, 41 cases), 3-hydroxy-3-methylglutaryl CoA lyase deficiency (3-HMGD, 4 cases), neonatal intrahepatic cholestasis caused by citrin deficiency (NICCD, 3 cases), citrullinemia type I (CIT-I, 8 cases), hypermethioninemia (HMET, 9 cases), 2-methylbutyryl-CoA dehydrogenase deficiency (2-MBDD, 4 cases), medium-chain acyl-CoA dehydrogenase deficiency (MCADD, 3 cases), glutaric acidemia type II (GA-II, 1case), ornithine transcarbamoylase deficiency (OTCD, 1 case), and argininemia (HARG, 1 case) (Figure 2). Other IMDs were not found in our study.

### 3.2 ML system model vs. the reference interval in the initial screening

To assess the effectiveness of the ML system model, this study utilizes extensive neonatal screening data. Initially, we analyzed the positive rate between the ML system model and pediatricians using a predefined reference interval in the initial screening process. The findings indicate that, based on the reference interval, 3,608 newborns received a positive diagnosis, while 305,494 cases were categorized as negative during the initial screening phase, resulting in a positive rate of 1.17%. On the other hand, the ML system model identified 1,029 positive cases and 308,073 negative cases in the initial screening, representing a positive rate of 0.33%. The results showed that the positive rate of initial screening significantly decreased from 1.17% to 0.33% compared with the results achieved by the reference interval. This could minimize false positives in the initial screening that cause unnecessary family stress as well as potentially enable cost-effective screening.

### 3.3 Diagnostic efficiency of ML system model for suspected positive cases

To interpret the potential of ML in enhancing diagnostic efficiency, we employed data from 180 suspected positive cases with NGS results. The application of ML system model diagnosed 142 true-positives (TPs) and 22 true-negatives (TNs). Compared with the reference interval, 144 TPs and 2 TNs were identified. In addition, the application of ML system model detected 6 FPs and 10 FNs, while the utilization of reference interval detected 26 FPs and 8 FNs.

The application of ML system model significantly reduced the number of FPs from 26 to 6, and the detailed numbers of TNs, TPs, FNs, and FPs were presented in Table 2. The sensitivity of the ML system model and the reference interval was 93.42% and 94.74%, respectively. The specificity of the ML system model was 78.57%, while that of the reference interval was 7.14%. Furthermore, the ML system exhibited a positive predictive value (PPV) of 95.95% and a negative predictive value (NPV) of 68.75%, whereas the reference interval yielded a PPV of 84.71% and an NPV of 20%. The ML system model showed a significant increase in both PPV and NPV. The ML system exhibited a higher accuracy. The values of sensitivity, specificity, PPV, NPV, and accuracy were summarized in Table 3.

**Table 2:** Comparison of diagnostic performance metrics between the ML system model and reference interval in suspected cases.

Methods	TNs (*n*)	TPs (*n*)	FNs (*n*)	FPs (*n*)	Sensitivity (%)	Specificity (%)	PPV (%)	NPV (%)	Accuracy (%)
ML system model	22	142	10	6	93.42	78.57	95.95	68.75	91.11
Reference interval	2	144	8	26	94.74	7.14	84.71	20	81.11

ML, machine learning; TNs, true-positives; TPs, true-negatives; FNs, false-negatives; FPs, false-positives; PPV, positive predictive value; NPV, negative predictive value.

**Table 3:** Diagnosis and false negative rates of different IMDs: comparison between ML system and reference interval for neonatal screening.

IMDs	ML system model		Reference interval		Total
	High risk (*n *= 142)	False negative rate (%)	Positive (*n *= 144)	False negative rate (%)	
PAHD	20	13.04	20	13.04	23
BH4D	1	0	1	0	1
PCD	30	0	29	3.33	30
MMA	6	0	5	16.67	6
SCADD	8	0	8	0	8
IBDD	9	0	9	0	9
3-MCCD	38	7.32	40	2.44	41
3-HMGD	4	0	4	0	4
NICCD	3	0	2	33.33	3
CIT I	8	0	7	12.50	8
HMET	6	33.33	9	0	9
2-MBDD	4	0	4	0	4
MCADD	2	33.33	3	0	3
GA II	1	0	1	0	1
OTCD	1	0	1	0	1
HARG	1	0	1	0	1

ML, machine Learning; PAHD, phenylalanine hydroxylase deficiency; BH4D, tetrahydrobiopterin deficiency; PCD, primary carnitine deficiency; MMA, methylmalonic acidemia; SCADD, short-chain acyl-CoA dehydrogenase deficiency; IBDD, Isobutyryl-CoA dehydrogenase deficiency; 3-MCCD, 3-methylcrotonyl-CoA carboxylase deficiency; 3-HMGD, 3-hydroxy-3-methylglutaryl CoA lyase deficiency; NICCD, neonatal intrahepatic cholestasis caused by citrin deficiency; CIT-I, citrullinemia type I; HMET, hypermethioninemia; 2-MBDD, 2-methylbutyryl-CoA dehydrogenase deficiency; MCADD, medium-chain acyl-CoA dehydrogenase deficiency; GA-II, glutaric acidemia type II; OTCD, ornithine transcarbamoylase deficiency; HARG, argininemia.

### 3.4 Comparative analysis of ML system model and reference interval reveals misdiagnosis in suspected inherited metabolic disease cases

Different disorders showed varying diagnostic results when using the ML system model (Table 3). BH4D, PCD, MMA, SCADD, IBDD, NICCD, CPT I, 2-MBDD, GA II, OTCD, and HARG accurately predicted positive cases. Nonetheless, a comprehensive assessment revealed 10 instances of misdiagnosis. There were 3 cases of PAHD, 3 cases of 3-MCCD, 3 cases of HMET, and 1 case of MCADD, and the FN rates were 13.04%, 7.32%, 33.33%, and 33.33%, respectively. In the reference interval, a total of 8 cases of misdiagnosis were found, including 3 cases of PAHD, 1 case of PCD, 1 case of MMA, 1 case of 3-MCCD, 1 case of NICCD, and 1 case of CIT I, and the FP rates were 13.04%, 3.33%, 16.67%, 2.44%, 33.33% and 12.50%, respectively (Table 3). Remarkably, two cases of PAHD were missed by both methods. Table 4 presents an overview of all of 16 misdiagnosed cases and their metabolic indices. Among them, 3 cases were identified with homozygous or compound heterozygous mutations, and the remaining 13 cases were heterozygous carriers. This result suggests that heterozygous carriers are easily misdiagnosed regardless of whether the ML method or the reference interval scheme is used for analysis. It is important to note that misdiagnosis occurs mainly among heterozygous carriers. In some heterozygous carriers, metabolic alterations were observed despite a low-risk designation by the ML system. In contrast, in other heterozygous carriers, indicators of normal metabolism were classified as high risk by the ML system.

**Table 4:** The misdiagnosis results and genetic analysis for IMDs.

IMDs	Cases	ML system	Reference interval	Zygosity	Genetic analysis
PAHD	Case 1	High risk	Normal (PHE = 78.66 μmol)	Heterozygous	*PAH*, c.1197A>T (p.V399V)
	Case 2	Low risk	Abnormal (PHE = 103.02 μmol/L)	Compound heterozygous	*PAH*, c.1315 + 6T>A; c.158G>A (p.R53H)
	Case 3	Low risk	Normal (PHE = 54.05 μmol/L)	Heterozygous	*PAH*, c.527G>A (p.R176Q)
	Case 4	Low risk	Normal (PHE = 56.19 μmol/L)	Heterozygous	*PAH*, c.510T>A (p.H170Q)
PCD	Case 5	High risk	Normal (C0 = 9.79 μmol/L)	Homozygous	*SLC22A5*, c.1400C>G (p.S467C)
MMA	Case 6	High risk	Normal (C3 = 3.96 μmol/L)	Homozygous	*MUT*, c.1663G>A (p.A555T)
3-MCCD	Case 7	Low risk	Abnormal (C5OH = 0.56 μmol/L)	Heterozygous	*MCCC1*, c.1331G>A (p.R444H)
	Case 8	Low risk	Abnormal (C5OH = 0.53 μmol/L)	Heterozygous	*MCCC1,* c.639 + 2T>A (p.S164Rfs*3)
	Case 9	Low risk	Abnormal (C5OH = 0.54 μmol/L)	Heterozygous	*MCCC1*, c.1681 + 34T>A
	Case 10	High risk	Normal (C5OH = 0.48 μmol/L)	Heterozygous	*MCCC1*, c.1679dup (p.N560Kfs*10)
NICCD	Case 11	High risk	Normal (CIT = 24.69 μmol/L)	Heterozygous	*SLC25A13*, c.775C>T (p.Q259*)
CIT I	Case 12	High risk	Normal (CIT = 28.14 μmol/L)	Heterozygous	*ASS1*, c.207_209del (p.E71del)
HMET	Case 13	Low risk	Abnormal (MET = 55.49 μmol/L)	Heterozygous	*MAT1A*, c.1070C>T (p.P357l)
	Case 14	Low risk	Abnormal (MET = 51.7 μmol/L)	Heterozygous	*MAT1A*, c.1070C>T (p.P357l)
	Case 15	Low risk	Abnormal (MET = 63.0 μmol/L)	Heterozygous	*MAT1A*, c.315C>A (p.N105K)
MACCD	Case 16	Low risk	Abnormal (C10:1 = 0.28 μmol/L	Heterozygous	*ACADM*, c.709-1G>A
			C6 = 0.18 μmol/L		
			C8 = 0.34 μmol/L		
			C8:1 = 0.6 μmol/L		
			C10 = 0.3 μmol/L)		

ML, machine Learning; PAHD, phenylalanine hydroxylase deficiency; PCD, primary carnitine deficiency; MMA, methylmalonic acidemia; 3-MCCD, 3-methylcrotonyl-CoA carboxylase deficiency; NICCD, neonatal intrahepatic cholestasis caused by citrin deficiency; HMET, hypermethioninemia; MCADD, medium chain acyl-CoA dehydrogenase deficiency; PHE, phenylalanine; CIT, citrulline; MET, Methionine; C0, free carnitine; C3, propionylcarnitine; C5OH, 3-hydroxy (OH) isovalerylcarnitine; C6, hexanoylcarnitine; C8, octanoylcarnitine; C8:1, octenoylcarnitine; C10, decanoylcarnitine.

### 3.5 Diagnostic acccuracy and alterations in metabolic indicators in patients with homozygous and compound heterozygous mutations vs. heterozygous carriers

The results of the present study revealed a remarkable diagnostic accuracy of 97.22% in a cohort of 72 patients with homozygous and compound heterozygous mutations, as assessed by the ML system. Similarly, the ML system demonstrated a diagnostic accuracy of 88.75% for 80 heterozygous carriers. Importantly, the metabolic indicators revealed significant differences between patients harboring homozygous mutations and those harboring compound heterozygous mutations compared with heterozygous carriers. Patients with IMDs harboring homozygous mutations and compound heterozygous mutations exhibited more significant alterations in metabolic indicators.

Table 5 provides a comprehensive overview of the diagnostic outcomes and alterations in metabolic indicators identified in patients with IMDs harboring homozygous and compound heterozygous mutations, as well as heterozygous carriers. This table presents detailed insights into the diagnostic efficacy and metabolic changes within these populations.

**Table 5:** Ml system true positive rates, and metabolic indicators in homozygous/compound heterozygous and heterozygous carriers for inherited metabolic disorders.

IMDs	Zygosity	ML system TP rate	Metabolic indicators
PAHD	Homozygous/compound heterozygous	93.75% (15/16)	PHE (966.45 ± 618.33 μmol/L)
	Heterozygous carriers	71.43% (5/7)	PHE(270.15 ± 256.90 μmol/L)
BH4D	Heterozygous carriers	100% (1/1)	PHE(196.22 μmol/L)
PCD	Homozygous/compound heterozygous	100% (14/14)	C0 (6.98 ± 1.73 μmol/L)
	Heterozygous carriers	100% (16/16)	C0 (8.84 ± 9.44 μmol/L)
MMA	Homozygous/compound heterozygous	100% (5/5)	C3 (5.96 ± 1.96 μmol/L)
	Heterozygous carriers	100% (1/1)	C3 (4.38 μmol/L)
SCADD	Homozygous/compound heterozygous	100% (5/5)	C4 (0.94 ± 0.30 μmol/L)
	Heterozygous carriers	100% (3/3)	C4 (0.79 ± 0.08 μmol/L)
IBDD	Homozygous/compound heterozygous	100% (8/8)	C4 (1.10 ± 0.25 μmol/L)
	Heterozygous carriers	100% (1/1)	C4 (0.70 μmol/L)
3-MCCD	Homozygous/compound heterozygous	100% (12/12)	C5OH (4.35 ± 4.44 μmol/L)
	Heterozygous carriers	89.66% (26/29)	C5OH (1.52 ± 1.99 μmol/L)
3-HMGB	Heterozygous carriers	100% (4/4)	C5OH (0.87 ± 0.33 μmol/L)
NICCD	Homozygous/compound heterozygous	100% (3/3)	CIT (38.01 ± 22.54 μmol/L)
CIT-I	Homozygous/compound heterozygous	100% (3/3)	CIT (120.82 ± 20.39 μmol/L)
	Heterozygous carriers	100% (5/5)	CIT (42.42 ± 9.79 μmol/L)
HMET	Homozygous/compound heterozygous	100% (1/1)	MET (375.44 μmol/L)
	Heterozygous carriers	62.5% (5/8)	MET (91.4 ± 62.59 μmol/L)
2-MBDD	Homozygous/compound heterozygous	100% (4/4)	C5 (1.43 ± 1.00 μmol/L)
MCADD	Homozygous/compound heterozygous	100% (2/2)	C6 (0.34 ± 0.04 μmol/L)
			C8 (1.69 ± 0.57 μmol/L)
	Heterozygous carriers	0% (0/1)	C6 (0.18 μmol/L)
			C8 (0.34 μmol/L)
GAII	Homozygous/compound heterozygous	100% (1/1)	C6 = 0.2 μmol/L
			C8 = 0.29 μmol/L
			C10 = 0.57 μmol/L
			C12 = 0.87 μmol/L
			C14 = 0.45 μmol/L
			C14:1 = 0.41 μmol/L
			C14:2 = 0.07 μmol/L
OTCD	Homozygous/compound heterozygous	100% (1/1)	CIT = 2.47 μmol/L
HARG	Heterozygous carriers	100% (1/1)	ARG = 93.93 μmol/L

## 4 Discussion

In our study, a total of 309,102 newborns underwent screening, leading to the identification of 152 cases with IMDs. The incidence of IMDs with our study population was 1: 2,034, a finding that consistently mirrors the reported rates in Suzhou (1: 3,163) (14) and Quanzhou (1: 2,804) (15). Notably, the most frequent IMDs within our cohort included PADH and 3-MCCD, exhibiting incidence rates of 1 in 13,429 and 1 in 7,539, respectively. It is pertinent to acknowledge that the incidence rates of IMDs exhibit substantial variations when examined across diverse geographical regions.

Recent research endeavors have witnessed the application of ML methodologies in the study of IMDs, albeit often focusing on individual or limited categories of IMDs (16). For instance, Zaunseder et al. (17) endeavored to enhance specificity in neonatal screening for IVA through the adept utilization of machine learning techniques. Similarly, Zhu et al. (18) pioneered the development of a machine learning logistic regression analysis model aimed at ameliorating the diagnostic accuracy associated with Phenylketonuria. Furthermore, Peng et al. (19) conducted an insightful analysis of the Random Forest machine's performance concerning GA-I, MMA, OTCD, and VLCADD. In contrast, our investigation adopts a holistic approach, harnessing the potential of nine distinct algorithms that collectively empower our ML system model to predict and scrutinize 31 distinct IMDs across a broad spectrum. This comprehensive strategy not only extends the scope of applicability but also augments the clinical value of our study, promising multifaceted insights into IMDs diagnosis and management.

It is imperative to acknowledge that ML techniques commonly exhibit enhanced predictive capabilities when applied to substantial datasets (20). In our study, the ML system showcased a notable diminished the initial positive screening rate in contrast to the traditional reference interval. These observations not only signify a reduction in the count of suspected cases but also hold promise for curtailing the necessity of patient recall.

In our investigation, ML model was successfully deployed, accumulating data from the Ningbo region. The experimental outcomes furnish compelling evidence substantiating the effectiveness of the ML system in the diagnosis of IMDs via neonatal screening. In direct comparison with the traditional reference interval, our ML system exhibited a sensitivity level akin to that of 93.42%, thereby maintaining diagnostic acumen. However, it notably improved specificity, elevating it from a mere 7.14% to a substantial 78.57%. This enhancement translated into a noteworthy reduction in the number of FPs, diminishing their count from 26 to a mere 6. This achievement is in alignment with prior research conducted by Peng et al. (19).

Furthermore, our ML system demonstrated a markedly heightened PPV of 95.95%, outperforming the traditional reference interval which yielded a PPV of 84.71%. These findings are congruent with the work of Zhu et al. (18), who reported a notable increase in the PPV for PAHD through the application of an ML model, surging from 19.14% to an impressive 32.16%. The substantial reductions in FPs and the concurrent augmentation of PPV underscore the significant improvements in screening efficiency realized through the application of ML methodologies.

However, in the relentless pursuit of augmented diagnostic precision and efficiency, it becomes imperative to address and comprehend the emergence of FNs, a pivotal concern inherent to any diagnostic framework. In the work of Tang et al. (21), four instances of NICCD were erroneously overlooked, signifying the susceptibility to FN outcomes. Similarly, Lin et al. (22) reported the misdiagnosis of a MADD patient whose acylcarnitine levels resided within the normal reference range upon recall. Within our findings, it's noteworthy that our ML model exhibited the omission of 10 cases, while the traditional reference interval missed 8 cases. The ML system model demonstrated an elevated false negative rate for 3-MCCD, HMET, and MCADD in comparison to the reference interval.

To elucidate the underpinnings of these FNs within the ML system model, we conducted a comprehensive analysis encompassing genetic results and metabolite concentrations for the missed cases. Within the cohort of cases missed by the ML model, which includes 3 cases of HMET, 3 cases of 3-MCCD, 3 cases of PAHD, and 1 case of MCADD, all cases, except for one PAHD case harboring a compound heterozygous mutation in PAH, were identified as heterozygous carriers of these conditions. This observation suggests a proclivity for heterozygous carriers to be susceptible to misclassification by the ML system model, emphasizing the intricacies of carrier identification within diagnostic frameworks.

This study stands as an innovative endeavor in the realm of medical science, pioneering the application of ML techniques for the precise diagnosis of patients afflicted with IMDs harboring pathogenic mutations. Among the cohort of 80 patients classified as heterozygous carriers, the ML system displayed a remarkable capacity by accurately identifying 71 cases, thus underscoring its consistent performance across a spectrum of diverse metabolic indices. It is imperative to note that heterozygous carriers, particularly those bearing partially functional alleles, exhibited discernible variations in metabolic profiles when compared to cases characterized by classical mutations (23–25). The presence of a single mutated allele induced noteworthy alterations in associated proteins and enzymes, thereby engendering variations in pertinent metabolic markers. A compelling observation emerged, revealing that the concentration of metabolites in heterozygous carriers registered a significant reduction when juxtaposed with patients harboring homozygous or compound heterozygous mutations. This revelation has profound implications for heterozygous carriers, particularly within the domain of neonatal screening practices as administered by pediatricians. Our findings provide cogent evidence to posit that heterozygous carriers may manifest variations in metabolic indicators, thereby precipitating potential misclassification. To surmount this challenge, it becomes imperative to embark on further research endeavors aimed at the refinement and augmentation of ML algorithms, with a specific emphasis on enhancing the capacity for accurate carrier detection.

The differences in metabolite profiles observed in heterozygous carriers, especially in PAHD patients, may affect gene expression due to variations in noncoding regions or deep introns, thereby forming compound heterozygous mutations affecting gene function, which also highlights the limitations of targeted region capture sequencing in the detection of noncoding regions and deep intron regions. These undetected mutations significantly affected metabolic profiles, suggesting that genetic interactions are more complex than previously understood and that the availability of NGS could make whole-exome and whole-genome sequencing more affordable and compensate for the lack of targeted region capture. In order to cope with these limitations, ML has become an indispensable supplement. Its ability to analyze large data sets, including metabolic profiles and clinical measures, improves predictive accuracy and reduces diagnostic errors. Newborn screening could be more effective by combining the pattern recognition and prediction capabilities of machine learning with the genetic insights provided by NGS.

To address the risk of missed diagnoses in newborns with initially normal screening results, as well as those born prematurely or with low birth weight, our institution has implemented a seven-year follow-up program. This method is aimed at maximizing the detection of late-onset symptoms of metabolic disorders that may not be evident in initial screenings. Specifically, this long-term monitoring ensures that children who may show symptoms of IMDs are promptly identified and assessed, significantly reducing the risk of missed diagnoses. By adopting this comprehensive follow-up, our study addresses the concerns regarding the limitations of DBS acquisition. The follow-up program was guided by the ‘Expert Consensus on Chinese Pediatric Health Examination’ to monitor and ensure the health of newborns (26). Two key assessments were initiated within the first month after discharge, and during the first year, children underwent quarterly examinations that included a history review, physical examination, physical measurements (e.g., height, weight, body-mass index, and head circumference), laboratory and imaging studies, and cognitive, neuromotor, developmental, language, hearing, vision, and dental health. Subsequently, the follow-up period was a biannual assessment, which was gradually changed to annual. Children with an increased risk of metabolic diseases due to genetic factors should be examined and evaluated intensively.

An intriguing case in our study involved a patient with MMA who harbored a homozygous mutation of the MUT gene c.1663G>A, despite no abnormal changes in metabolic indicators (C3 = 3.96 μmol/L). This highlights that certain IMDs may not manifest obvious changes in MS/MS neonatal screening during the early stages, resulting in FN results. As more cases of this nature are unveiled, the capability of our ML system to identify asymptomatic cases will be increasingly evident, emphasizing its potential toimprove the detection of such cases.

Furthermore, ML algorithms typically benefit from large datasets, as larger datasets theoretically result in improved predictive performance. As our model continues to be implemented in Ningbo, we anticipate that the utilization and advancement of ML algorithms will gain increasing popularity in the near future. This trend is expected to further enhance the prediction accuracy and computational performance of risk assessment models for neonatal IMDs.

## 5 Conclusions

In conclusion, the application of our ML system exhibited promising effectiveness in pediatric diagnostic screening of IMDs. The model achieved a sensitivity of 93.42% and a specificity of 78.57%, surpassing the performance of the reference interval. Furthermore, the ML system demonstrated increased PPV and NPV. Notably, the ML system proved to be valuable in identifying carrier patients, providing novel insights into the application of ML in pediatric medical practice for diagnosing IMDs through neonatal screening.

## Data Availability

The raw data supporting the conclusions of this article will be made available by the authors without further reservation. The datasets generated and/or analysed during the current study are not publicly available due to regulation of Women and Children's Hospital of Ningbo University and protecting patient personal information. Requests to access these datasets should be directed to the corresponding author HL, Email: lihaibo-775@163.com.
