# Supplementary material for: The significance of machine learning in neonatal screening for inherited metabolic diseases
Source: Front Pediatr. 2024 Mar 20;12:1366891. doi: 10.3389/fped.2024.1366891 (PMC10993727; doi:10.3389/fped.2024.1366891)
Supplement: Supplementary file 1 [file Table1.docx]

Supplementary Material

The Significance of Machine Learning in Neonatal Screening for Inherited Metabolic Diseases

Xiangchun Yang, Shuxia Ding, Jianping Zhang, Zhuojie Hu, Danyan Zhuang, Fei Wang, Shanshan Wu, Changshui Chen^*^ , Haibo Li^*^

*** Correspondence:** Changshui Chen: [chencs@139.com](mailto:chencs@139.com); Haibo Li: lihaibo-775@163.com

1. **Supplementary Tables**

**Supplementary Table 1.** Abbreviations and reference interval for acylcarnitines and amino acids in the study.

| Analytes | Abbreviations | Reference interval (μmol/L) |
| --- | --- | --- |
| Free Carnitine | C0 | 9.5-57 |
| Acetylcarnitine | C2 | 4-48.3 |
| Propionylcarnitine | C3 | 0.42-4.5 |
| Malonylcarnitine | C3DC | 0.02-0.44 |
| Butyrylcarnitine | C4 | 0.08-0.51 |
| 3-hydroxy（OH）isovalerylcarnitine | C5OH | 0.07-0.48 |
| Isovalerylcarnitine | C5 | 0.04-0.41 |
| Tiglylcarnitine | C5:1 | 0-0.05 |
| Glutarylcarnitine | C5DC | 0.02-0.35 |
| Hexanoylcarnitine | C6 | 0.02-0.17 |
| Adipylcarnitine | C6DC | 0.04-0.28 |
| Octanoylcarnitine | C8 | 0.01-0.17 |
| Octenoylcarnitine | C8:1 | 0.02-0.43 |
| Decenoylcarnitine | C10:1 | 0.01-0.27 |
| Decadienoylcarnitine | C10:2 | 0.01-0.08 |
| Decanoylcarnitine | C10 | 0.02-0.21 |
| Dodecenoylcarnitine | C12:1 | 0.01-0.23 |
| Dodecanoylcarnitine （Lauroyl） | C12 | 0.02-0.28 |
| Tetradecadienoylcarnitine | C14:2 | 0.01-0.06 |
| Myristoleylcarnitine （Tetradecenoyl） | C14:1 | 0.01-0.24 |
| Myristoylcarnitine （Tetradecanoyl） | C14 | 0.05-0.4 |
| 3-hydroxy（OH）myristoylcarnitine | C14OH | 0.00-0.06 |
| Hexadecenoylcarnitine | C16:1 | 0.02-0.49 |
| Palmitoylcarnitine | C16 | 0.37-6.60 |
| 3-hydroxy（OH）palmitoleylcarnitine | C16:1OH | 0.01-0.1 |
| 3-hydroxy（OH）palmitoylcarnitine | C16OH | 0.01-0.06 |
| Linoleylcarnitine | C18:2 | 0.05-071 |
| Octadecenoylcarnitine （Oleyl） | C18:1 | 0.27-2.93 |
| Octadecanoylcarnitine （Stearoyl） | C18 | 0.12-1.8 |
| 3-hydroxy（OH）octadecenoylcarnitine （3-OH-oleyl） | C18:1OH | 0.01-0.07 |
| 3-hydroxy（OH）octadecanoylcarnitine （3-OH-stearoyl） | C18OH | 0.01-0.04 |
| Alanine | ALA | 135-797 |
| Valine | VAL | 57-317 |
| Glycine | GLY | 203-1200 |
| Ornithine | ORN | 42.7-380 |
| Arginine | ARG | 1.66-50.27 |
| Leucine | LEU | 74.63-322 |
| Methionine | MET | 9.16-41 |
| Phenylalanine | PHE | 27.79-100 |
| Tyrosine | TYR | 36.17-299 |
| Citrulline | CIT | 6.32-37 |
| Proline | PRO | 98.25-512 |
| Succinylacetone | SA | 0-2.0 |
